# A multicenter controlled trial on knowledge and attitude about cardiopulmonary resuscitation among secondary school children in Malaysia

**DOI:** 10.1186/1865-1380-6-37

**Published:** 2013-10-17

**Authors:** Nik Hisamuddin NA Rahman, Chew Keng Sheng, Tuan Hairulnizam T Kamauzaman, Abu Yazid Md Noh, Shaik Farid A Wahab, Ida Zarina Zaini, Mohd Hashairi Fauzi, Andey Ab Rahman, Nur Shahidah Dzulkifli

**Affiliations:** 1 Department of Emergency Medicine, School of Medical Sciences, Kubang Kerian 16150, Malaysia

## Abstract

**Background:**

We performed a multicenter controlled trial to assess the knowledge and attitude (KA) about cardiopulmonary resuscitation (CPR) among secondary school children in a district in Malaysia.

**Methods:**

This was a prospective intervention study. The primary endpoint of the study was to determine the level of KA about resuscitation after CPR training. The six schools and classes from selected schools were chosen by randomization among the form three and four classes using sealed envelopes. A fully validated questionnaire consisting of three sections (sociodemographic, knowledge and attitude) was given to the pupils before and 2 weeks after the intervention. The intervention group was given a lecture, video show, pamphlet and 1-h practical session on CPR training. The control group received a placebo in order to overcome the learning effect. The maximum scores for the knowledge and attitude sections were 72 and 28, respectively. Repeated measures ANOVA analysis was used for specific objectives to determine the changes in knowledge and attitude level pre- and post-intervention for both study groups. *P*-values less than 0.05 were taken as significant at 95% confidence intervals.

**Results:**

The mean (SD) total knowledge scores for the intervention (*n* = 216) and control (*n* = 252) groups were 62.43 (13.68) and 62.29 (12.11), respectively (maximum score 72) (*p* > 0.05). On the other hand, the mean (SD) total attitude scores for the intervention and the control groups were 19.33 (4.51) and 17.85 (4.52), respectively (maximum score 28) (*p* < 0.001). There were significant differences in mean knowledge and attitude scores between the intervention and control groups with regard to time (pre- and post-intervention). The mean difference in knowledge and attitude scores between both study groups was 8.31 (*p* < 0.001) and 2.39 (*p* < 0.001), respectively.

**Conclusions:**

The level of knowledge and attitudes of secondary school children was shown to be acceptable prior to the intervention. Furthermore, a brief CPR training program improved their level of knowledge and attitudes significantly as compared to those who had never been trained.

## Background

Cardiopulmonary resuscitation (CPR) is a series of life-saving actions that improves the chance of survival following cardiac arrest [1]. The purpose of CPR is to temporarily provide effective oxygenation to the vital organs, especially the brain and heart, through artificial circulation of oxygenated blood until the restoration of normal cardiac and respiratory activity takes place [2]. Historically, Dr. Peter Safar, also known as “the father of CPR,” together with James Elam was the first to demonstrate the effectiveness of CPR through an experiment. In the 1970s, it was first promoted as a technique that the public should learn in the USA [3]. Out-of-hospital cardiac arrest (OHCA) is a significant public health problem. It affects all communities worldwide. However, recognition of cardiac arrest is not always straightforward, especially for laypersons. Therefore, the survival rates among OHCA victims are still low [4]. Many studies have shown that early bystander CPR is one of the most important factors for survival from OHCA. In the current situation, only about 20-30% of adults with OHCA receive bystander CPR [5,6].

In 2001, an International Liaison Committee of Resuscitation (ILCOR) symposium on education in resuscitation strongly recommended the inclusion of CPR in school curriculums as an invaluable long-term investment. The suitable and appropriate environment in school makes it an easy place for training and reinforcement of knowledge and skills. School children are more accessible and more easily motivated than adults. They learn quickly and retain skills well. CPR training for school students was first introduced in Norway as early as the 1960s [7]. Since then, it has been pursued in many other countries, including Canada, Denmark, Poland and several states in the USA [8,9]. In 2001, the author of the Malaysian Clinical Practice Guidelines on Acute Myocardial Infarction strongly urged that teaching of CPR needs to be extended to the general public as well [10].

Unfortunately, no previous studies have ever been conducted in Malaysia to analyze the knowledge and attitude concerning CPR among secondary school children. For this reason, we set out to determine the effectiveness of CPR training on knowledge and attitude about BLS among secondary school children in the district of Kota Bharu, Malaysia. We targeted secondary school children because they are highly motivated and able to provide effective chest compression, particularly at the ages of 13 to 15 years old [11,12]. The study was approved by the Ministry of Education Malaysia and the medical school ethics board and committee. The objective of the study was to determine the effectiveness of CPR training on knowledge and attitude (KA) about resuscitation among secondary school children.

## Methods

**Figure 1:**
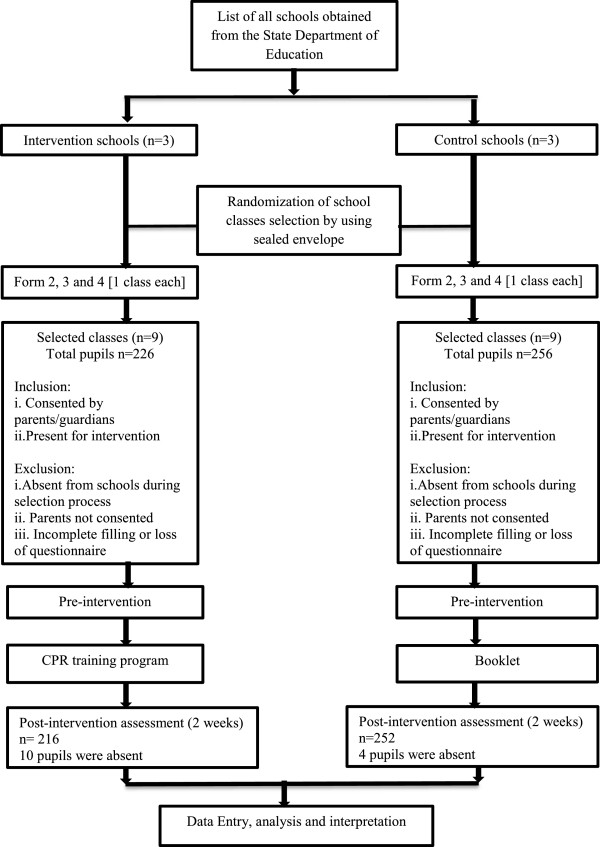
Study flow chart.

This was a prospective intervention study. The primary endpoint of the study was to determine the level of KA about resuscitation before and after CPR education among secondary school children in a district in Kota Bharu, Malaysia. This study was conducted for a 6-month period from January 2012 to June 2012. Six schools were chosen to be part of the study. Three schools were selected as a control group and the other three as the intervention group. The schools and classes from selected schools were chosen by randomization among form three and four classes using sealed envelopes (Figure 1). The inclusion and exclusion criteria included:

### Inclusion criteria

•All randomly selected from form two, three and four secondary school children.

•Written consent given by parents or guardian.

### Exclusion criteria

•Students who were absent from school on the selection day.

•Parents or guardians who did not consent to have their children participate in this study.

•Boarding school pupils.

•Special school (schools for disabled, etc.).

A questionnaire was developed to objectively assess the effects of the interventions. The questionnaire was fully validated by the principal investigator 6 months prior to the study for content and face validation. The reliability test was carried out using Cronbach’s analysis. The validation and reliability tests were carried out by Emergency Medicine Specialists and tested on 220 pupils in two secondary schools that were not included in the study. The validated questionnaire was analyzed by the local statistician. The questionnaire, which consists of three sections (sociodemographic, knowledge and attitude), was given to the pupils before and after the intervention (Tables 1 and 2). For the second section on knowledge, the question consists of 18 “very sure,” “sure,” “don’t know,” “unsure” and “very unsure” statements reflecting knowledge related to heart disease, risk factors for cardiac arrest and CPR. The items were given score of four for very sure, three for sure, two for don’t know, one for unsure and zero for very unsure. The total possible maximum score on the knowledge domain was 72.

**Table 1:** Variables asked about in the knowledge section of the validated questionnaire

**Knowledge**	**Answer**				
**About cardiac arrest:**	**Very sure**	**Sure**	**Don’t know**	**Unsure**	**Very unsure**
Cardiac arrest is due to arterial occlusion					
Central chest pain is a sign of cardiac arrest					
Shortness of breath is a warning sign of cardiac arrest					
Cardiac arrest can be treated					
Healthy lifestyle reduces the risk of cardiac arrest					
**Risk factor of heart attack:**	**Very sure**	**Sure**	**Don’t know**	**Unsure**	**Very unsure**
Old age					
Stress					
Smoking					
Hypertension					
Hyperlipidemia					
Alcohol					
Diabetes mellitus					
Overweight					
Lack of exercise					
**CPR (2010 Guidelines):**	**Very sure**	**Sure**	**Don’t know**	**Unsure**	**Very unsure**
“C-A-B” stands for circulation-airway-breathing					
Rate of compression is 100/min					
To check for response, gently tap the victim’s shoulder and call the patient with a loud voice.					
Give two mouth-to-mouth rescue breaths					

**Table 2:** Variables asked about in the attitude section in the validated questionnaire

**Attitude**	**Strongly agree**	**Agree**	**Not sure**	**Disagree**	**Strongly disagree**
You are a 65 year old with underlying hypertension and hyperlipidemia and suddenly have a heart attack leading to cardiac arrest. Do you want CPR performed on you?					
You are a 75 year old who has had a stroke and heart attack, and had second heart attack leading to cardiac arrest. Do you want CPR performed on you?					
You are an 85 year old, very ill with underlying advanced cancer and heart failure, and suddenly have cardiac arrest. Do you want CPR performed on you?					
You are a 90 year old, healthy person who suddenly has cardiac arrest. Do you want CPR performed on you?					
Are you willing to perform mouth-to-mouth ventilation on your family members?					
Are you willing to perform mouth-to-mouth ventilation on an unknown person?					
Do you think teaching school children CPR/BLS is appropriate?					

The third section on attitude consists of seven statements. The first four statements are about various scenarios with collapsed patients. The respondents were asked whether CPR should be performed or not in each scenario. The last three statements were about performing mouth-to-mouth ventilation (MMV) among family members, teaching BLS in school and teaching BLS to the public. Responses to the statements were “strongly agree,” “agree,” “not sure,” “disagree” and “strongly disagree.” The items were given a score of four for strongly agree, three for agree, two for not sure, one for disagree and zero for strongly disagree. A total possible maximum score in the attitude domain was 28. The entire scoring system was developed according to advice from local statisticians.

Since no studies have used this questionnaire, a preliminary study was done prior to the actual study in order to calculate the sample size. Power sampling (PS) software way applied using *t*-tests for independent samples to determine the sample size. The estimated difference in the mean knowledge score between the intervention and control groups was selected based on the standard deviation (SD) and effect size. The SD was taken from preliminary study (knowledge score control group), and 0.50 was selected as the value for effect size. The minimum required sample size with a power of the study of 0.85 was 252 per group including the 20% dropout factor.

The intervention group was given a lecture, video show, pamphlet and practical session on CPR training. The contents of the lecture given include the definition of sudden cardiac arrest, information on heart disease, risk factors for cardiac arrest, information on bystander CPR as well as the chain of survival. Information regarding CPR skill was mainly taught during the practical session. The lecture was delivered in the Malaysian language for 15 min. The content of the lecture is very basic introductory information for school children on heart disease and cardiac arrest. The lecture was short to avoid boredom among the pupils and to encourage them to listen attentively. A video demonstrating CPR was made by the researcher and team based on the video produced by the 2010 AHA Guidelines on CPR. For the practical session on CPR training, we prepared 20 manikins for respondents to use and one manikin for the main facilitator. A facilitator was present for each manikin to assist the students during the practical session. Our facilitators were all certified in 2010 BLS by St John Ambulance of Malaysia, Civil Defense of Malaysia: nurses, medical assistants and medical students who volunteered to be involved in this study. All of the facilitators were briefed about the CPR teaching protocol in order to standardize the CPR training among the school children. The control group received a placebo in order to overcome the learning effect. The respondents were given a booklet on the hazard of smoking. It was five pages long and presented in the Malaysian language. The booklet was provided as a courtesy from the Unit of Biostatistics and Research Methodology of the study center. A control group was required in order to prove the effectiveness of the simplified and brief education on CPR and heart disease (intervention) to show that the results were not just due to pure chance.

The same questionnaire was given out to the same pupils 2 weeks after the initial CPR training. The 2-week duration until the repeat questionnaire distribution was decided on based upon discussion with the statistician, and we also wanted to do it sooner in order to obtain a more reliable result on effects post-intervention. The data were analyzed using SPSS, version 18.0.1, licensed to the medical school. Chi-square test was used mainly for descriptive study involving sociodemographic data as well as baseline knowledge and attitude prior to the intervention. Repeated measures ANOVA analysis was used for specific objectives to determine the changes in the knowledge and attitude levels pre- and post-intervention for both study groups. A *P*-value of less than 0.05 was taken as significant at a 95% confidence interval.

## Results

There were a total of 477 questionnaires distributed among the selected secondary school children from six different schools in Kota Bharu district. The study comprised 226 respondents in the control group and 256 respondents in the intervention group during the pre-intervention study. However, during the post-intervention study visit, four respondents from the control group and ten respondents from the intervention group were absent. The numbers of the final intervention and control respondents were 216 and 252, respectively. The response rate was 98% for the control group and 96% for the intervention group. Table 3 shows a demographic comparison between the two study groups.

**Table 3:** Distribution of sociodemographic characteristics of respondents for the control and intervention groups

**Variables**	**Control ( *n *** **=226)**	**Intervention**	***p*-value **^**a**^
	***n *** **(%)**	**(*n *** **= 251)**	
		***n *** **(%)**	
Gender			<0.001
Male	137 (60.6)	73 (29.1)	
Female	89 (39.4)	178 (70.9)	
Form			0.356
Two	85 (37.6)	93 (37.1)	
Three	75 (33.2)	97 (38.6)	
Four	66 (29.2)	61 (24.3)	
Father’s occupation			<0.001
Professionals	68 (30.1)	87 (34.7)	
Non-professionals	21 (9.3)	75 (29.9)	
Others	137 (60.6)	89 (35.5)	
Mother’s occupation			0.035
Professionals	52 (23.0)	78 (31.1)	
Non-professionals	6 (2.7)	13 (5.2)	
Others	168 (74.3)	160 (63.7)	
School category			0.086
Normal daily	165 (73.00)	165 (65.7)	
Cluster	61 (27.0)	86 (34.3)	
CPR training			0.027
Yes	59 (26.1)	89 (35.5)	
No	167 (73.9)	162 (64.5)	
Duration of CPR training			0.092
<6 months	9 (15.3)	10 (11.2)	
6-12 months	17 (28.8)	14 (15.7)	
1-2 years	15 (25.4)	21 (23.6)	
>2 years	18 (30.5)	44 (49.4)	
CPR facilitator			0.391^b^
Healthcare provider	3 (5.1)	2 (2.3)	
Non-healthcare	56 (94.9)	86 (97.7)	
provider			

^a^Pearson’s chi-Square test.

^b^Fisher’s exact test.

There were significant differences between these two groups in terms of experience in CPR training. The majority of the study subjects in the control group 73.9% (167) and intervention group 64.5% (162) had no CPR training in this study. On the other hand, only 26.1% (59) from the control group and 35.5% (89) from the intervention group had experienced in CPR training. Overall, more students in the intervention group had exposure to CPR training than in the control group. There was no significant difference (*p* = 0.092) between these two groups in terms of duration of CPR training. Out of 59 study subjects from the control group who had experience in CPR training, 15.3% [9] of them had learned CPR for less than 6 months, 28.8% [13] between 6 and 12 months, 25.4% [14] between 1 and 2 years, and 30.5% [15] for more than 2 years. For the intervention group, out of 89 students who had experience in CPR training, 15.3% [9] had learned CPR for less than 6 months, 28.8% [13] between 6 and 12 months, 25.4% [14] between 1 and 2 years, and 30.5% [15] for more than 2 years. Among those respondents who had past experience in CPR training, only three respondents in the control group and two respondents in the intervention group had received training from healthcare providers. The rest of the respondents had learned CPR from non-healthcare providers, for example, school teachers and members of St John Ambulance.

There was no significant statistical difference in the baseline (before interventions) knowledge score between the intervention and control groups [62.43 (13.68) and 62.29 (12.11) respectively; *p* = 0.902]. However, there was a slight different in the baseline attitude score for both groups [(19.33 (4.51) and 17.85 (4.52) respectively; *p* < 0.001]. The changes/increments (pre- and post-intervention) in the knowledge and attitude scores among the intervention group pupils were 17.79 (CI: 16.32, 19.26) and 2.84 (CI: 2.35, 3.33), respectively. However, there was much less change/increment (pre- and post-intervention) in the knowledge and attitude scores among the control group pupils, 0.99 (CI: 0.39, 1.58) and 0.89 (CI: 0.67, 1.11), respectively. Table 4 and Table 5 show the detailed distribution of responses for both the knowledge and attitude sections. Table 6 shows a comparison of the actual pre and post mean knowledge and attitude scores on resuscitation in the intervention and control group based on time. The increments of knowledge score in the the intervention and control groups were 28.6% and 1.6%, respectively. Similarly, the increment in the positive attitude score was higher in the intervention group compared to the control group (14.7% vs. 4.9%, respectively).

**Table 4:** Distribution of questions and responses in the knowledge and attitude section

**No.**	**Knowledge**	**Control (*n *** **= 226) [n (%)]**					**Intervention (n=251) [n (%)]**				
		**Very sure**	**Sure**	**Don’t know**	**Unsure**	**Very unsure**	**Very sure**	**Sure**	**Don’t know**	**Unsure**	**Very unsure**
**About cardiac arrest:**											
1.	Cardiac arrest is due to arterial occlusion	40 (17.7)	69 (30.5)	43 (19.0)	70 (31.0)	4 (1.8)	71 (28.3)	66 (26.3)	55 (21.9)	49 (19.5)	10 (4.0)
2.	Central chest pain is a sign of cardiac arrest	21 (9.3)	81 (35.8)	49 (21.7)	64 (28.3)	11 (4.9)	24 (9.6)	73 (29.1)	62 (24.7)	79 (31.5)	13 (5.2)
3.	Shortness of breath is a warning sign of cardiac arrest	35 (15.5)	91 (40.3)	46 (20.4)	45 (19.9)	9 (4.0)	55 (21.9)	69 (27.5)	51 (20.3)	66 (26.3)	10 (4.0)
4.	Cardiac arrest can be treated	32 (14.2)	63 (27.9)	42 (18.6)	82 (36.3)	7 (3.1)	41 (16.3)	66 (26.3)	64 (25.5)	73 (29.1)	7 (2.8)
5.	Healthy lifestyle reduces the risk of cardiac arrest	150 (66.4)	47 (20.8)	13 (5.8)	14 (6.2)	2 (0.9)	187 (74.5)	26 (10.4)	14 (5.6)	15 (6.0)	9 (3.6)
**Risk factor of heart attack:**											
6.	Old age	44 (19.5)	64 (28.3)	45 (19.9)	53 (23.5)	20 (8.8)	66 (26.3)	69 (27.5)	38 (15.1)	65 (25.9)	13 (5.2)
7.	Stress	46 (20.4)	66 (29.2)	44 (19.5)	62 (27.4)	8 (3.5)	67 (26.7)	51 (20.3)	55 (21.9)	64 (25.5)	14 (5.6)
8.	Smoking	94 (41.6)	71 (31.4)	23 (10.2)	33 (14.6)	5 (2.2.)	113 (45.0)	65 (25.9)	27 (10.8)	38 (15.1)	8 (3.2)
9.	Hypertension	47 (20.8)	60 (26.5)	51 (22.6)	57 (20.2)	11 (4.9)	70 (27.9)	66 (26.3)	56 (22.3)	48 (19.1)	11 (4.4)
10.	Hyperlipidemia	96 (42.5)	54 (23.9)	48 (21.2)	28 (12.4)	0 (0.0)	113 (45.0)	71 (28.3)	37 (14.7)	25 (10.0)	5 s(2.0)

**Table 5:** Distribution of statements and responses in the attitude section

**No.**	**Attitude**	**Control (*n *** **= 226) [*n *** **(%)]**					**Intervention (n=251) [n (%)]**				
		**Strongly agree**	**Agree**	**Not sure**	**Disagree**	**Strongly disagree**	**Strongly agree**	**Agree**	**Not sure**	**Disagree**	**Strongly disagree**
1.	You are 65 years old with underlying hypertension and hyperlipidemia and suddenly have aheart attack leading to cardiac arrest. Do you want CPR performed on you?	41 (18.1)	78 (34.5)	77 (34.1)	19 (8.4)	11 (4.9)	101 (21.2)	168 (35.2)	157 (32.9)	25 (5.2)	26 (5.5)
2.	You are a 75 year old who has had a stroke and heart attack, and had second heart attack leading to cardiac arrest. Do you want CPR performed on you?	45 (19.9)	64 (28.3)	72 (31.9)	27 (11.9)	18 (8.0)	55 (21.9)	83 (33.1)	70 (27.9)	29 (11.6)	14 (5.6)
3.	You are 85 years old, very ill with underlying advanced cancer and heart failure, and suddenly have cardiac arrest. Do you want CPR performed on you?	37 (16.4)	46 (20.4)	84 (37.2)	25 (11.1)	34 (15.0)	56 (22.3)	63 (25.1)	66 (26.3)	43 (17.1)	23 (9.2)
4.	You are a 90-year-old, healthy person who suddenly has cardiac arrest. Do you want CPR performed on you?	42 (18.6)	65 (28.8)	76 (33.6)	27 (11.9)	16 (7.1)	61 (24.3)	88 (35.5)	60 (23.9)	22 (8.8)	20 (8.0)
5.	Are you willing to perform MMV on your family members?	74 (32.7)	74 (32.7)	67 (29.6)	9 (4.0)	2 (0.9)	111 (44.2)	65 (25.9)	57 (22.7)	10 (4.0)	8 (3.2)
6.	Do you think teaching school children CPR/BLS is appropriate?	50 (22.1)	78 (34.5)	61 (27.0)	19 (8.4)	18 (8.0)	106 (42.2)	69 (27.5)	57 (22.7)	12 (4.8)	7 (2.8)
7.	Do you agree that CPR/BLS should be taught to the public?	74 (32.7)	82 (36.3)	56 (24.8)	10 (4.4)	4 (1.8)	115 (45.8)	71 (28.3)	51 (20.3)	5 (2.0)	9 (3.6)

**Table 6:** Comparison of knowledge and attitude scores on resuscitation between the two groups based on time using RMA^*^

**Variables**	**Time**	**Group**	**Mean score**	**95% CI**	***p*-value**
Knowledge	Pre	Control	62.29	(60.58, 64.01)	*P* = 0.902
		Intervention	62.20	(60.56, 63.84)	
	Post	Control	63.28	(61.51, 65.05)	*P* < 0.001
		Intervention	79.99	(78.30, 81.69)	
Attitude	Pre	Control	17.83	(17.23, 18.43)	*P* = 0.001
		Intervention	19.25	(18.67, 19.82)	
	Post	Control	18.72	(18.13, 19.31)	*P* = 0.001
		Intervention	22.09	(21.52, 22.65)	

*Repeated measures ANOVA (RMA) analysis was applied.

## Discussion

Coronary artery disease is the most common cause of death worldwide. In Malaysia, cardiovascular disease remains the leading cause of death, accounting for 20%−25% of all death in a government hospital from 2000–2011 [11]. Cardiac arrest is the most disastrous outcome following a cardiovascular event. A recent study by Chew et al. in a state on the East Coast of Malaysia showed that only 3% of all the out-of-hospital cardiac arrest (OHCA) patients actually survived to hospital discharge [12]. This worrying figure is attributed to many local factors, such as the low rate of public knowledge about CPR and poor EMS services. There are no local data on the rate of public performance on CPR. Likewise, no actual figure indicates the knowledge about public-assisted defibrillation. The average ambulance response time varies among cities, depending on the quantity of traffic on the road and the size of the city. The average ambulance response time is 18 min in the locality of the study center [16]. Even though the ambulance personnel are taught on how to use the automated external defibrillator, unfortunately we do not have the actual figure on the rate of its use nationwide. Immediate commencement of CPR has been highlighted as crucial for surviving cardiac arrest, especially bystander CPR, as many cardiac arrest cases occur at home and in public places [17].

We visualized that teaching school children CPR is perhaps the best opportunity and method to educate the public about this lifesaving technique, and this has been supported by many studies [14,18]. However, in Malaysia we do not have any exact scientific figures on the level of knowledge and attitude (KA) about CPR among secondary school children. The rating of KA about CPR among the public is one of the crucial indicators of the potential outcome of OHCA. We asked the respondents about their knowledge related to heart attacks, risk factors of heart attacks and, importantly, theoretical knowledge on CPR as well as their attitude about different cardiac emergency scenarios before we commenced the CPR teaching program. Pre-intervention, we found that more than 80 percent of the respondents from both study groups were “very sure/sure” that a healthy lifestyle reduces the risk of heart attack. However, few were aware that heart attack can be treated and central chest pain is a cardinal sign of a heart attack. Out of nine statements regarding cardiac arrest risk factors, more than two-thirds of the respondents in both study groups were “very sure/sure” that lack of exercise, smoking, hyperlipidemia and being overweight were risk factors for a heart attack. Diabetes mellitus (DM) was chosen as the factor with the least risk by the respondents from both study groups. One study was done in London regarding public perception and experiences of myocardial infarction, cardiac arrest and CPR. From that study, they concluded that the majority of the public was aware that chest pain was the most likely recognized symptom of heart attack [13]. The high positive response rate may be attributed to easily accessible information on diseases through either the Internet or media. With the advancement of the technology, school pupils can simply search for information regarding heart attacks and CPR [15,19]. Overall, more students wanted CPR performed on them, especially those in the intervention group as compared to the control group. However, we did not further discuss factors affecting attitudes about performing CPR in this study.

The simplified CPR teaching program has significantly increased the knowledge and attitude scores of the respondents with regards to time as shown in the repeated measure ANOVA analysis. There was a significant difference in the mean knowledge and attitude scores between the intervention and control groups. From this analysis, we found that the scores were markedly higher in the intervention group as compared to the control group. Similarly, in 2007, a study conducted by Connolly et al. on teaching basic life support in school children revealed that the pupils who were trained in CPR showed a highly significant increase in their level of knowledge following the training session [20]. In addition, they were also able to perform CPR adequately as compared to those who had never been trained. CPR training for secondary school children should include sufficient practical training to obtain the required CPR skills standards. Recently, a study was conducted to determine whether a combination of CPR training with e-learning could be an effective method for students from different educational level groups. They concluded that a combination of 1 h of e-learning and 1 h of practical training following a 2-h refresher course is a good alternative for better outcomes. However, the skills obtained were also dependent on the students’ levels of education [21]. It is hard for us to recommend a definite period for the CPR refresher course for the public for a variety of reasons, such as personal motivation, commitment, willingness and attitude. Ideally for the non-healthcare providers, a 2-yearly refresher course would be ideal and has been applied to first aid and first responder courses as well. The findings in this study have also provided useful baseline information for future intervention studies. One factor affecting the good outcome in this study is the mode of delivering the message. The Malay language as the national language was used throughout the CPR training program. Thus, this was considered to improve the message penetration. Another factor contributing to the effectiveness of CPR training on knowledge and attitude is choosing the right program for the right target group [22]. Even though our current CPR training program has not been tested yet in the Malaysian general population, the outcome of this study has proven that the current training program used is very appropriate for school children and also for the rest of the community. Now, we have to move forward. The need is not only for more bystander CPR, but also for better quality CPR. Good theoretical skills and attitudes about CPR do not ensure an increase in willingness to perform CPR. Besides teaching techniques, we must also address the motivation of every single learner, hoping they can translate their knowledge and attitude into better perception regarding BLS from one generation to another.

Despite the strengths, a few limitations were identified in this study. There were slight differences in the distribution of gender, family background and previous training experience in CPR between the two study groups, but these factors were not considered crucial in the statistical analysis. However, the number of those without CPR training experience prior to the intervention was equal between the two groups. Self-administered questionnaires are vulnerable to subject reporting bias such as recall bias, which results from inaccurate recall of the previous intervention program. Respondents might not spend time giving reliable and unbiased views of their knowledge and attitudes. In order to prevent bias, verification of responses from the respondents should have been done. However, due to limitations of time and manpower, this could not be done. We also found a few missing values or non-responses from the respondents on the self-administered questionnaires, especially during the early part of the study. To avoid this from happening, the researcher and team ensured each of the questions was answered before the respondents turned in the questionnaires. For the intervention part, it was delivered only once in a short period of time to match with the students’ schedules. The current CPR training program used in this study had not been previously tested elsewhere, especially in the Malaysian population. No further evaluation on the effectiveness of the module (lecture, self-instructed video on CPR demonstration and pamphlet) was done.

## Conclusion

It is shown that the levels of knowledge and attitudes about CPR among secondary school children were acceptable prior to the intervention. Furthermore, following the CPR training program, their level of knowledge and attitudes had improved significantly compared to those who had never been trained. Finally, we have proven to the higher authorities that we should start teaching school children CPR now for a better tomorrow.

## Competing interests

The authors declare that they have no competing interests.

## Authors’ contributions

NH prepared the proposal for the study, involved in the data collection and manuscript preparation. TH, CKS, AY, IZ, SF, AR involved in the data collection. NS participated in the ethical submission, data collection and data analysis. All authors read and approved the final manuscript.

## References

[B1] Field JM American Heart Association Guidelines for Cardiopulmonary Resuscitation and Emergency Cardiovascular Care Science Circulation 2010 6 S640 S656 10.1161/CIRCULATIONAHA.110.970889 20956217

[B2] Tintinalli EJ Emergency Medicine: A Comprehensive Study Guide (6th Edition) 2003 United States: McGraw Hill 120 121

[B3] Mitka M Safar PJ JAMA 2003 6 19 2485 2486 12759308 10.1001/jama.289.19.2485

[B4] Berdowski J Berg RA Tijssen JGP Koster RW Global incidences of out-of-hospital cardiac arrest and survival rates: Systematic review of 67 prospective studies Resuscitation 2010 6 11 1479 1487 10.1016/j.resuscitation.2010.08.006 20828914

[B5] Bohm K Rosenqvist M Herlitz J Hollenberg J Svensson L Survival is similar after standard treatment and chest compression only in out-of-hospital bystander cardiopulmonary resuscitation Circulation 2007 6 25 2908 2912 10.1161/CIRCULATIONAHA.107.710194 18071077

[B6] Graesner JT Wnent J Bein B Doerges VJ Impact of bystander CPR on the outcome of patients after pre-hospital cardiac arrest Resuscitation 2008 6 Supplement(0 S45

[B7] Naqvi S Siddiqi R Hussain SA Batool H Arshad H School children training for basic life support J Coll Phys Surg Pakistan 2011 6 10 611 615 10.2011/JCPSP.611615 22015122

[B8] Liberman M Golberg N Mulder D Sampalis J Teaching cardiopulmonary resuscitation to CEGEP students in Quebec—a pilot project Resuscitation 2000 6 3 249 257 10.1016/S0300-9572(00)00236-7 11114454

[B9] Reder S Cummings P Quan L Comparison of three instructional methods for teaching cardiopulmonary resuscitation and use of an automatic external defibrillator to high school students Resuscitation 2006 6 3 443 10.1016/j.resuscitation.2005.08.020 16678958

[B10] Robaayah D Clinical Practice Guidelines on Acute Myocardial Infarction 2001 National Heart Institute Kuala Lumpur, Malaysia: Ministry Of Health Malaysia CPG

[B11] Acute Coronary Syndrome (ACS) Registry - Leading the Charge for National Cardiovascular Disease (NCVD) Database National Cardiovascular Disease Database http://www.malaysiaheart.org 19230244

[B12] Chew KS Yazid MN The willingness of final year medical and dental students to perform bystander cardiopulmonary resuscitation in an Asian community Int J Emerg Med 2008 6 4 301 309 10.1007/s12245-008-0070-y PMC2657260 19384646

[B13] Donohoe RT Haefeli K Moore F Public perceptions and experiences of myocardial infarction, cardiac arrest and CPR in London Resuscitation 2006 6 1 70 79 10.1016/j.resuscitation.2006.03.003 16945467

[B14] Plant N Taylor K How best to teach CPR to schoolchildren: a systematic review Resuscitation 2013 6 4 415 421 10.1016/j.resuscitation.2012.12.008 23246989

[B15] Jelinek GA Gennat H Celenza T O'Brien D Jacobs I Lynch D Community attitudes towards performing cardiopulmonary resuscitation in Western Australia Resuscitation 2001 6 3 239 246 10.1016/S0300-9572(01)00411-7 11738773

[B16] Anisah A Chew KS Mohd Shaharuddin Shah CH Nik Hisamuddin NA Patients' perception of the ambulance services at Hospital Universiti Sains Malaysia Singapore Med J 2008 6 8 631 635 18756347

[B17] Kumar S Ewy GA The hospital's role in improving survival of patients with out of-hospital cardiac arrest Clin Cardiol 2012 6 8 462 466 10.1002/clc.21992 22549822 PMC6652703

[B18] Hill K Mohan C Stevenson M McCluskey D Objective assessment of cardiopulmonary resuscitation skills of 10-11-year-old schoolchildren using two different external chest compressions to ventilation ratios Resuscitation 2009 6 1 96 99 10.1016/j.resuscitation.2008.08.005 18952356

[B19] Kuramoto N Morimoto T Kubota Y Maeda Y Seki S Takada K Hiraide A Public perception of and willingness to perform bystander CPR in Japan Resuscitation 2008 6 3 475 481 10.1016/j.resuscitation.2008.07.005 18805615

[B20] Connolly M Toner P Connolly D McCluskey D The 'ABC for life' programme—teaching basic life support in schools Resuscitation 2007 6 2 270 10.1016/j.resuscitation.2006.06.031 17134814

[B21] Cuijpers P Kicken W Gorgels A Effectiveness of a blended learning approach for CPR training in secondary schools for different education level groups Resuscitation 2012 6 Supplement 1(0) e112

[B22] Lafferty C Larsen P Galletly D Resuscitation teaching in New Zealand schools J N Z Med Assoc 2003 6 1181 U582 14581965

